# Supplementary material for: Higher Virulence of Diplodia seriata Isolates on Vines of cv. Cabernet Sauvignon Associated with 10-Year-Old Wood Compared to Young Tissue
Source: Plants (Basel). 2023 Aug 18;12(16):2984. doi: 10.3390/plants12162984 (PMC10459257; doi:10.3390/plants12162984)
Supplement: Supplementary file 1 [file plants-12-02984-s001.zip › Supplementary Table S3.pdf]

Table S3. Isolates of *D. seriata* and *D. mutila*, obtained from GenBank Database included in this study for phylogenetic analysis.

Species	Strain	Host	Reference	GenBank accession number[a]	
				ITS	BT
Diplodia seriata	CBS112555	*Vitis vinifera*	Alves *et al*., 2004	AY259094	DQ458856
	UCD244Ma	*Vitis vinifera*	Úrbez-Torres and Gubler, 2009	DQ008314	DQ008337
	UCD352Mo	*Vitis vinifera*	Úrbez-Torres and Gubler, 2009	DQ008315	DQ008338
	UCD614Tu	*Vitis vinifera*	Úrbez-Torres and Gubler, 2009	DQ008318	DQ008341
	UCD710SJ	*Vitis vinifera*	Úrbez-Torres and Gubler 2009	DQ008321	DQ008344
	USD770St	*Vitis vinifera*	Úrbez-Torres *et al*., 2008	DQ008322	DQ008345
	UCD1010BC	*Vitis vinifera*	Úrbez-Torres *et al*., 2008	EU012377	EU012429
	UCD1015BC	*Vitis vinifera*	Úrbez-Torres *et al*., 2008	EU012378	EU012430
	UCD1035BC	*Vitis vinifera*	Úrbez-Torres *et al*., 2008	EU012379	EU012431
	UCD1038BC	*Vitis vinifera*	Úrbez-Torres *et al*., 2008	EU012380	EU012432
	UCD1052BC	*Vitis vinifera*	Úrbez-Torres *et al*., 2008	EU012381	EU012433
	UCD1061BC	*Vitis vinifera*	Úrbez-Torres *et al*., 2008	EU012382	EU012434
Diplodia mutila	PUCV 1493	*Vitis vinifera*	Larach et al., 2020		
	PUCV 1510	*Vitis vinifera*	Larach et al., 2020		
	PUCV 1556	*Vitis vinifera*	Larach et al., 2020		
	PUCV 1568	*Vitis vinifera*	Larach et al., 2020		
	PUCV 2174	*Vitis vinifera*	Larach et al., 2020		

	PUCV 2177	*Vitis vinifera*	Larach et al., 2020		
	UCD288Ma	*Vitis vinifera*	Urbez-Torres *et al*., 2006	DQ008313	DQ008336
	UCD1953SB	*Vitis vinifera*	Urbez-Torres *et al*., 2006	DQ233598	DQ233619
	UCD1965SB	*Vitis vinifera*	Urbez-Torres *et al*., 2006	DQ233599	DQ233620
Neofusicoccum parvum	CBS110301	*Vitis vinifera*	Alves *et al*., 2004	AY259098	EU673095

[a] ITS = internal transcribed spacer region; BT = β-tubulin gene.
